# The Prevalence and Predictors of Sickle Cell Anemia in the Saudi Arabia General Population: Findings from a Cross-Sectional Study

**DOI:** 10.3390/healthcare13101117

**Published:** 2025-05-11

**Authors:** Naif M. Alhawiti, Mamdouh M. Shubair, Amani Alharthy, Badr F. Al-Khateeb, Raed Aldahash, Bandar Aleissa, Khadijah Angawi, Mohammed AlJumah, Afaf Almutairi, Maram N. Almutairi, Sumera A. Ali, Ashraf El-Metwally

**Affiliations:** 1 Department of Clinical Laboratory Sciences, College of Applied Medical Sciences, King Saud bin Abdulaziz University for Health Sciences, Riyadh 11481, Saudi Arabia; hawitin@ksau-hs.edu.sa; 2 King Abdullah International Medical Research Center, Riyadh 21423, Saudi Arabia; khateebb@ngha.med.sa (B.F.A.-K.); dr.raaed@hotmail.com (R.A.); elmetwally.ashraf@outlook.com (A.E.-M.); 3 School of Health Sciences, Faculty of Human and Health Sciences (FHHS), University of Northern British Columbia (UNBC), 3333 University Way, Prince George, BC V2N 4Z9, Canada; 4 Department of Health Science, College of Health and Rehabilitation Sciences, Princess Nourah bint Abdulrahman University, Riyadh 11671, Saudi Arabia; afalharthy@pnu.edu.sa; 5 Department of Epidemiology and Biostatistics, College of Public Health & Health Informatics, King Saud bin Abdulaziz University for Health Sciences, Riyadh 11481, Saudi Arabia; 6 King Abdulaziz Medical City, Ministry of National Guard-Health Affairs, Riyadh 11426, Saudi Arabia; 7 Department of Medicine, Ministry of National Guard-Health Affairs, Riyadh 11426, Saudi Arabia; 8 Department of Medicine, King Saud bin Abdulaziz University for Health Sciences, Riyadh 11481, Saudi Arabia; 9 Department of Health Systems and Quality Management, College of Public Health & Health, King Saud bin Abdulaziz University for Health Sciences, Riyadh 11481, Saudi Arabia; bandarmd@gmail.com; 10 Department of Health Services and Hospital Administration, Faculty of Economics and Administration, King Abdulaziz University, Jeddah 21589, Saudi Arabia; kkangawi@kau.edu.sa; 11 King Fahd Medical City (KFMC), Ministry of Health (MOH), Riyadh 11525, Saudi Arabia; jumahm@gmail.com (M.A.); maramnaif95@gmail.com (M.N.A.); 12 Dr. Sulaiman Al Habib Medical Group, Riyadh 12987, Saudi Arabia; afafalmutairi11@gmail.com; 13 Riyadh Second Health Cluster, Ministry of Health (MOH), Riyadh 11461, Saudi Arabia; 14 Department of Pediatrics, University of Alberta, Edmonton, AB T6G 2R3, Canada; saziz2@ualberta.ca; 15 College of Public Health and Health Informatics, King Saud bin Abdulaziz University for Health Sciences, Riyadh 11481, Saudi Arabia

**Keywords:** sickle cell anemia, prevalence, predictors, Saudi Arabia cross-sectional study

## Abstract

**Background/Objectives**: Despite the high incidence of sickle cell anemia in Saudi Arabia, little is known about the sociodemographic characteristics, behavioral risk factors, and concomitant conditions of the condition. We performed this study to measure the prevalence of sickle cell anemia and its associated predictors among Saudi residents. **Methods**: This cross-sectional study was conducted in 48 primary healthcare centers across Saudi Arabia. A total of 14,239 Saudi residents were included through multi-stage random sampling. Data on sociodemographic variables, behavioral factors, and comorbidities were collected using a validated and reliable questionnaire. Univariate and multivariate logistic regression analyses were performed to identify the predictors of sickle cell anemia, with the statistical significance set at a *p*-value of <0.05. All analyses were carried out using SPSS version 26 for Windows. **Results**: Overall, the prevalence of sickle cell anemia was found to be 3.2% among Saudi residents. There was a positive association between insurance coverage and sickle cell anemia (AOR: 1.87; 95% CI: 1.52, 2.31). The odds of sickle cell anemia were 1.39 times higher among diabetic than non-diabetic individuals (AOR: 1.39; 95% CI: 1.01, 1.91). There were positive associations between sickle cell anemia and hypertension (AOR: 1.70; 95% CI: 1.23, 2.35), high cholesterol (AOR: 2.38; 95% CI: 1.74, 3.24), and heart disease (AOR: 8.05; 95% CI: 6.05, 10.71). **Conclusions**: Our findings indicate significant associations between sickle cell anemia and insurance coverage, smoking, obesity, diabetes mellitus, hypertension, hypercholesterolemia, and heart disease. While the overall prevalence of sickle cell anemia in our study was relatively modest, the Saudi Arabian government should prioritize the objective quantification of the disease burden across the population to effectively mitigate its consequences.

## 1. Introduction

Sickle cell anemia, an autosomal recessive disease, represents a complex group of inherited blood disorders stemming from a mutation in the beta-globin gene, leading to the polymerization of hemoglobin S [1]. Although various forms of sickle cell disease exist, homozygous sickle cell anemia is the most prevalent and severe type [1]. Globally, sickle cell anemia stands out as the most common hemoglobin abnormality among various hemoglobinopathies, thus posing a significant public health concern [2]. Individuals with this multisystem disorder can experience a range of severe symptoms, including vaso-occlusive crises, chronic hemolytic anemia, gallstones, stroke, acute chest syndrome, and multiple organ failure [3,4]. While the complications of sickle cell anemia are often manageable, patients frequently require admission to intensive care units [3].

Sickle cell anemia is prevalent in sub-Saharan Africa, parts of India, the Caribbean, the Mediterranean region, and the Middle East [5]. In Africa, sickle cell anemia is considered the third primary cause of hospital admissions and mortality, following lower respiratory tract infections and malaria [6,7,8]. According to the Global Burden of Disease 2023 report, sub-Saharan Africa has experienced a significant increase in both sickle cell disease incidence and prevalence over the last two decades [9]. The number of births with sickle cell disease in the region has risen by 27.2%, and the all-age prevalence has surged by 67.4% during this period [9]. Similarly, in Arab countries, the large family sizes and high rates of consanguinity may make Arabs uniquely likely to undergo genetic analysis to detect inherited diseases such as sickle cell anemia [10]. Sickle cell anemia affects only 0.26% of Saudi population, however, about 4% of Saudi residents are found to have the sickle cell trait [11,12].

It has been reported that sickle cell anemia affects approximately 1.2% of the population in the eastern province of Saudi Arabia, with an additional 17% reported to carry the sickle cell trait [11,12]. Although the prevalence of sickle cell anemia has decreased across Saudi Arabia, the overall burden of the disease remains higher compared to other countries [13]. Furthermore, sickle cell anemia is considered endemic in the southern and eastern regions of Saudi Arabia [14]. Despite this significant burden, there is a paucity of data regarding the sociodemographic and behavioral risk factors and comorbidities associated with sickle cell anemia in the Saudi population. To address this knowledge gap, we conducted this study to assess the burden of sickle cell anemia and identify its associated predictors among Saudi residents.

## 2. Materials and Methods

### 2.1. Study Design, Study Duration, Study Setting, and Sampling Technique

A cross-sectional study was conducted from March to July 2023. To obtain a representative sample of individuals utilizing primary healthcare centers (PHCs) within the Riyadh region, a multi-stage cluster sampling method was employed. In the first stage, Riyadh was stratified into three health clusters. Health Cluster 2 was selected for this study due to its demographic diversity and well-established healthcare infrastructure, serving an estimated 3.7 million residents across 103 PHCs. In the second stage, a stratified random sampling technique was applied within Health Cluster 2 to select 48 PHCs. This stratification ensured the proportional representation of PHCs from both the urban and suburban areas within the cluster. Finally, within each of the 48 selected PHCs, eligible participants were identified and recruited using systematic random sampling. This multi-stage approach aimed to yield a representative sample of the general population accessing primary healthcare services in Riyadh Health Cluster 2, thereby minimizing potential selection bias and enhancing the generalizability of the findings to this specific region.

### 2.2. Eligibility Criteria and Target Population

#### 2.2.1. Inclusion Criteria

The study’s target population included all individuals over the age of 18 years who visited the selected primary healthcare centers in Riyadh during the study period, irrespective of their nationality (Saudi or non-Saudi) or residency status. Any person attending these primary healthcare centers as a visitor was eligible to participate in the survey.

#### 2.2.2. Exclusion Criteria

Individuals under the age of 18 years were not eligible to participate in this survey. Furthermore, healthcare professionals and employees of the primary healthcare facilities were also excluded from the study. Visitors to the primary healthcare center’s waiting room were contacted by a data collector to complete an electronic survey form. Lastly, study participants who did not provide informed consent were not included in the study.

### 2.3. Development and Description of Study Questionnaire

The study questionnaire was developed through a collaborative effort between the Central Health Services Reform Management Team and consultants representing diverse regions across Saudi Arabia. This initiative was part of a larger health system reform program focused on evaluating the health perceptions, behaviors, and priorities across all health clusters within the nation. The questionnaire was designed to capture a broad spectrum of factors influencing health outcomes and healthcare utilization. It was structured into several sections, encompassing self-reported health status, health priorities and concerns, and health-related behaviors such as smoking, fast food consumption, physical activity, and alcohol use. Furthermore, it assessed the health perceptions on a scale from excellent to poor, collected sociodemographic data (e.g., age, education level, employment status, sex, and marital status), and gathered detailed information on the medical history and comorbidities, including heart disease, diabetes, obesity, hypertension, and hypercholesterolemia. The questionnaire also included inquiries regarding insurance coverage and whether the participants had received a diagnosis of sickle cell anemia. This comprehensive design ensured the tool captured a diverse array of factors pertinent to understanding the population’s health and healthcare needs.

### 2.4. Validation and Reliability Assessment of Questionnaire

A rigorous evaluation process was implemented to ensure the questionnaire’s validity and reliability. The content validity was established through a review by a panel of 15 experts, including healthcare professionals and public health specialists, who assessed the questionnaire’s relevance, clarity, and appropriateness. Based on their feedback, certain items were modified or removed to enhance the tool’s effectiveness. The face validity was evaluated in a pilot study involving 200 participants who assessed the clarity, difficulty, and comprehensibility of the questions. Trained data collectors further facilitated understanding by reading the questions aloud during the interviews. The test–retest reliability was assessed by administering the questionnaire twice to 100 participants from the pilot study, with the second administration conducted via phone following the initial adjustments. The calculated test–retest reliability coefficient of 0.83 indicated high consistency and reliability. Furthermore, to ensure linguistic precision, the questionnaire was translated from English to Arabic and subsequently back-translated into English, maintaining the accuracy and integrity of the tool across both languages.

### 2.5. Pilot Study and Rationale for Selecting Hail City

The pilot study was conducted in Hail City, selected by the Central Health Services Reform Management Team as an ideal testing site due to its demographic and health profile, which closely reflects that of the broader Saudi population, making it a representative location for preliminary testing. This pilot phase involved 100 patients and 20 focus group participants who assessed the clarity, comprehensibility, and difficulty of the questionnaire items. During the focus group discussions, the participants identified unclear, difficult, or insufficiently explicit questions. These were subsequently revised or rephrased to ensure ease of understanding for the participants in the main study. The test–retest reliability was further evaluated by readministering the revised questionnaire via phone to the same 100 participants, yielding a high reliability coefficient (0.83). This, along with a respectable degree of face validity, confirmed the questionnaire’s robustness. These assessments were completed in January 2023, prior to the commencement of the main data collection in Riyadh in March 2023. To guarantee linguistic accuracy, the questions initially written in English were translated into Arabic and then back-translated into English, a meticulous process that ensured the questionnaire’s readiness for large-scale implementation in Riyadh and the other health clusters across Saudi Arabia.

### 2.6. Data Collection Using Study Questionnaire

The electronic survey was administered with an interviewer present. Initially, data collectors at the primary healthcare clinics in Riyadh, Saudi Arabia, utilized a questionnaire that was developed and digitally implemented on iPads or Android devices. Before inviting individuals to participate in this sickle cell anemia research, the data collectors assessed their eligibility, ensuring all participants were 18 years of age or older. Subsequently, the eligible individuals were approached, the study’s objectives were explained, and written informed consent was obtained prior to their participation. Completion of the survey was voluntary. The data collectors then administered the questionnaire to those who provided consent. The survey collected data on sociodemographic characteristics including age, gender, household size, marital status, education level, employment status, and general health status. Additionally, it gathered information on behavioral factors such as smoking, consumption of fast food or junk food, alcohol use, and physical activity or exercise, as well as the presence of comorbidities like hypertension, diabetes, obesity, and COPD. A total of 14,239 participants completed the survey and constituted the final sample size for this analysis.

### 2.7. Statistical Methods

Following the evaluation of the data distribution using histograms, we presented descriptive statistics for the variables. Continuous variables exhibiting a normal distribution, such as age, were reported as means with standard deviations (SDs). To further analyze the distribution of the participants across different age groups, the age variable was subsequently categorized. Categorical variables including insurance coverage, health status, marital status, work status, and education, were presented as frequencies and proportions. Given that the primary outcome was a binary variable (sickle cell anemia: Yes/No), we conducted a univariate logistic regression to assess the associations between the various independent factors and sickle cell anemia. All factors with a *p*-value below 0.25 in the univariate analysis were considered for inclusion in the multivariate logistic regression model. Multivariate logistic regression was then performed to identify the independent predictors of sickle cell anemia, using a significance threshold of *p* < 0.05. Following the multivariate logistic regression, we reported the adjusted odds ratios (AORs) along with their corresponding 95% confidence intervals (CIs) for the predictors of sickle cell anemia. All statistical analyses were performed using SPSS version 26 for Windows.

## 3. Results

### 3.1. Sociodemographic Characteristics of Study Participants at Primary Health Care Centers of Saudi Arabia

Table 1 shows the sociodemographic characteristics of the study participants visiting primary health care centers in Saudi Arabia. On average, the participants were 59.77 years old (±16.35 standard deviation (SD)). About half of the study participants were 50 to 75 years old. With respect to education, slightly more than half of the study participants (51.5%) reported acquiring higher education, and more than half (56.6%) were females. About a third of the study participants (65.3%) were married, and 51.4% were employed. Only one third of the study subjects (33.7%) reported their health as in excellent condition, and about a quarter of the participants (24.3%) had insurance coverage. The data showed that 27.7% were smokers, 60.7% were physically active, 5.2% were obese, 12.4% were diabetic, 11.1% were hypertensive, and about half (50.3%) reported unsafe driving behaviors. Only 3.2% of the study participants were found to have sickle cell anemia in this population, as shown in Table 1.

### 3.2. Sociodemographic Predictors of Sickle Cell Anemia: Findings of Univariate and Multivariate Analyses

Table 2 reveals the sociodemographic predictors of sickle cell anemia among the residents of Saudi Arabia vising primary health care centers in Riyadh. The findings of the multivariate analysis showed that older individuals of 50 to 75 years of age (AOR: 0.77; 95% CI: 0.62, 0.95) and 75 years and above (AOR: 0.62; 95% CI: 0.44, 0.86) were 23% and 38% less likely to suffer from sickle cell anemia than younger individuals. Similarly, higher education was found to be a protective factor against sickle cell anemia. Males were 47% less likely to suffer from sickle cell anemia than females (AOR: 0.53; 95% CI: 0.42, 0.65). There was a positive association between insurance coverage and sickle cell anemia (AOR: 1.87; 95% CI: 1.52, 2.31).

### 3.3. Behavioral Risk Factors and Comorbidities Associated with Sickle Cell Anemia: Findings of Univariate and Multivariate Analyses

Table 3 shows the behavioral risk factors and comorbidities associated with sickle cell anemia. We found a positive and strong relationship between smoking status and sickle cell anemia (AOR: 3.60; 95% CI: 2.74, 4.71). The final model (Model 3) showed that physical activity was also positively related to sickle cell anemia, albeit with a small magnitude (AOR: 1.63; 95% CI: 1.17, 2.28). Likewise, after adjusting for age, sex, behavioral risk factors, and other comorbidities, the odds of sickle cell anemia were higher by 86% among obese than non-obese residents. Similarly, we found positive associations between diabetes (AOR: 1.39; 95% CI: 1.01, 1.91), hypertension (AOR: 1.70; 95% CI: 1.23, 2.35), and high cholesterol (AOR: 2.38; 95% CI: 1.74, 3.24) and sickle cell anemia. Finally, the odds of sickle cell anemia were 8.05 times higher among individuals with heart disease than without heart disease (AOR: 8.05; 95% CI: 6.05, 10.71), as shown in Table 3.

## 4. Discussion

We conducted this cross-sectional survey to estimate the prevalence of sickle cell anemia and explore its associations with sociodemographic factors, behavioral risk factors, and other comorbidities. Our findings indicate that approximately 3.2% of Saudi residents in the study region have sickle cell anemia. This prevalence is consistent with previous research; for instance, a study by Jastaniah (2011) reported a prevalence of 2.6% among Saudi residents, a figure closely aligned with our results [11]. However, the prevalence of the sickle cell trait exhibits a wide range, reported to be between 2% and 27% in various studies. In contrast, Hazzazi et al. (2020) found a much higher prevalence of sickle cell anemia (22.6%) among patients admitted to a hospital [15]. The discrepancy between our study’s findings and those of Hazzazi et al. (2020) [15] can likely be attributed to the variations in the study methodologies and settings. Hazzazi et al. conducted their research in a hospital environment, which typically caters to a higher concentration of patients with more severe or acute conditions. In contrast, our study was a population-based survey conducted among community-dwelling residents of Saudi Arabia.

Our findings indicate that individuals with comorbidities such as diabetes, hypertension, high blood cholesterol, and obesity had a higher likelihood of having sickle cell anemia compared to those without these co-occurring conditions. Overall epidemiological trends suggest an increasing number of individuals with both the sickle cell trait and type 2 diabetes (T2D). This co-occurrence of the two chronic diseases is concerning because even though the sickle cell trait is sometimes considered benign, research has demonstrated that it can elevate the risk of diabetes complications, including stroke, chronic kidney disease, and end-stage renal failure [16]. In addition, the association of T2D with sickle cell anemia could further worsen such complications, which may require further research to understand the complexity of having these two different diseases simultaneously [17]. Furthermore, our study revealed an association between high blood pressure and sickle cell anemia. Existing evidence suggests a link between sickle cell anemia and pulmonary hypertension. For example, a study by Ataga et al. (2004) indicated a substantial prevalence of pulmonary hypertension (30%) among patients with sickle cell anemia [18]. Another study by Oguanobi (2010) presented contradictory findings, suggesting significantly lower diastolic blood pressures and mean arterial blood pressures, but higher pulse pressures among patients with sickle cell anemia [19]. These mixed results underscore the need for future studies to further explore the complex relationship between high blood pressure and sickle cell anemia.

Our study also indicated that males were less likely to have sickle cell anemia than females. This finding may be coincidental, as the incidence of sickle cell anemia, being an autosomal recessive disorder, is not typically sex-linked. However, sex differences have been reported in complications associated with sickle cell anemia. For instance, previous research suggests that severe complications such as infections and adverse cardiovascular outcomes are more commonly observed in males [20]. Males are also more likely to develop musculoskeletal disorders and acute chest syndrome than females [20]. In addition, males are more likely to experience pain crisis than females [20]—a finding that Masese et al. (2021) did not support [20]. While we did not explore the differences in these sickle cell anemia-related complications, the sex-related differences for the disease itself need to be further explored in the future.

Our cross-sectional study revealed a positive association between insurance coverage and sickle cell anemia, indicating a higher likelihood of the condition among insured individuals compared to the uninsured. Given the cross-sectional nature of our study, which precludes inferring causation, this association could be interpreted in two ways. Firstly, it is plausible that individuals diagnosed with sickle cell anemia may subsequently seek or obtain insurance coverage to mitigate the substantial costs associated with managing the disease and its potential adverse outcomes. Secondly, it is also important to consider that individuals without insurance may face significant economic barriers to accessing regular medical treatment and diagnostic services. This could lead to an undercount of the sickle cell anemia cases within the uninsured population in our healthcare-based study, potentially biasing our findings and suggesting a lower prevalence in this group than may actually exist in the broader community. Therefore, while our findings highlight an association, future longitudinal studies are necessary to establish the temporal relationship between insurance coverage and sickle cell anemia. Additionally, community-based studies that are not reliant on healthcare access are crucial to accurately determine the true prevalence of sickle cell anemia across all insurance strata.

### Strengths and Limitations and Future Perspective

This large-scale study provides valuable insights into the factors associated with sickle cell anemia among primary healthcare attendees in Riyadh. The identification of these predictors can inform the development of targeted strategies and resource allocation for the timely early detection of sickle cell anemia within the Saudi resident population. The primary strengths of the current study lie in its substantial dataset and its highly representative sample of participants, enabling the extrapolation of our findings to Saudi Arabia and regions with similar sociodemographic profiles. The use of multi-stage random sampling minimized the potential for self-selection bias, ensuring that every Saudi resident visiting a primary healthcare center had an equal opportunity to participate. Furthermore, the data collection through a comprehensive survey questionnaire allowed us to investigate a wide array of sociodemographic, behavioral, health-related, and comorbid characteristics potentially associated with sickle cell anemia. Finally, the use of a valid and reliable survey instrument reduced the likelihood of measurement errors across the variables.

However, several limitations warrant consideration. Firstly, the cross-sectional design of this electronic survey precludes the establishment of the temporal order and causal relationships between the identified predictors and sickle cell anemia. Future longitudinal studies employing cohort or case–control designs are necessary to elucidate the directionality of these associations and to determine the potential causal pathways. Secondly, while measures were taken to ensure participant privacy and anonymity, the potential for social desirability bias in self-reported behavioral lifestyle factors such as smoking and alcohol use cannot be entirely ruled out. Future research could benefit from incorporating objective measures or utilizing validated, less direct questioning techniques to minimize this bias. Thirdly, the prevalence of sickle cell anemia (3.2%) reported in this study is based on self-reports, which may lead to an underestimation of the true prevalence within the population. Individuals unaware of their condition or those who do not regularly access healthcare services may not have been captured. Future epidemiological studies employing objective diagnostic testing within community-based settings are crucial to obtain a more accurate estimation of the sickle cell anemia prevalence in Saudi Arabia. Furthermore, establishing a national registry for sickle cell disease would provide continuous, comprehensive data on the prevalence, incidence, and disease burden over time, informing evidence-based policies and intervention strategies.

Moving forward, future research should also explore the familial clustering of sickle cell anemia, given its genetic nature, to better understand its transmission patterns within the Saudi population. Additionally, investigating the socio-economic determinants of sickle cell anemia and its associated comorbidities could inform targeted interventions to reduce health disparities. Finally, research focused on the effectiveness of different screening and management strategies, including the implementation of universal newborn screening and integrated care pathways, is essential to optimize outcomes for individuals living with sickle cell anemia in Saudi Arabia.

## 5. Conclusions

The self-reported prevalence of sickle cell anemia among Saudi residents was relatively low in this study. However, it showed positive associations with several significant factors, including insurance coverage, smoking, obesity, diabetes mellitus, hypertension, hypercholesterolemia, and heart disease. These associations suggest the potential for a higher burden of comorbid conditions among individuals with sickle cell anemia, which could worsen their overall health outcomes. Conversely, older ages and higher education levels appeared to be protective factors against sickle cell anemia, possibly due to better health awareness, better access to care, or healthier lifestyle choices in these groups. Therefore, the Saudi Arabian government and relevant health authorities should proactively evaluate the true burden of sickle cell anemia across the population using objective measures.

### Targeted Policy Recommendations for Sickle Cell Anemia Mitigation in Saudi Arabia

Building upon the identified prevalence and associations, the Saudi Arabian government should consider the following specific, actionable steps: Firstly, given the protective association observed with older ages and higher education levels, targeted public health initiatives should focus on health literacy and awareness campaigns, particularly among younger populations and those with lower educational attainment. These campaigns should emphasize the genetic nature of the sickle cell trait and disease, the importance of premarital screening and genetic counseling services, and the benefits of early diagnosis and adherence to management protocols. Utilizing accessible and culturally sensitive communication channels is crucial for effective dissemination.

Secondly, to proactively address the higher odds of comorbidities such as diabetes, hypertension, high cholesterol, and heart disease in individuals with sickle cell anemia, the government should invest in establishing specialized multidisciplinary clinics or integrated care teams dedicated to the comprehensive management of these patients. These centers should offer coordinated care, including regular screenings for these complications from an early age, specialized dietary and lifestyle counseling tailored to individuals with sickle cell anemia and the associated conditions, and access to specialists in hematology, endocrinology, cardiology, and nephrology under one roof.

Thirdly, recognizing the 3.2% prevalence, the government should strengthen and expand existing newborn screening programs to ensure the universal early detection of sickle cell disease across all regions of Saudi Arabia. This should be coupled with robust follow-up mechanisms to connect the diagnosed infants and their families with specialized care centers, genetic counseling services, and educational resources from the earliest possible stage. This early intervention can significantly improve the long-term outcomes and quality of life for affected individuals.

## Figures and Tables

**Table 1:** Sociodemographic characteristics of study participants at primary health care centers of Saudi Arabia (n = 14,239).

Age; Mean ± SD	59.77	16.35
Age	n	%
<50 years	4848	34
50 to 75 years	6945	48.8
At least 75 years	2446	17.2
Education	n	%
Primary	572	4
Secondary	3937	27.6
Higher education	7336	51.5
Others	2394	16.8
Gender	n	%
Female	8062	56.6
Male	6177	43.4
Marital status	n	%
Not married	4939	34.7
Married	9300	65.3
Employment status	n	%
Employed	7317	51.4
Unemployed	6922	48.6
Health status	n	%
Excellent	4798	33.7
Very good	5076	35.6
Good	2815	19.8
Fair	1256	8.8
Poor	294	2.1
Insurance coverage	n	%
Yes	3457	24.3
No	10,782	75.7
Smoking	n	%
No	10,297	72.3
Yes	3942	27.7
Physical activity	n	%
No	5598	39.3
Yes	8641	60.7
Obesity	n	%
No	13,502	94.8
Yes	737	5.2
Diabetes	n	%
No	12,474	87.6
Yes	1765	12.4
Hypertension	n	%
No	12,659	88.9
Yes	1580	11.1
Driving behavior	n	%
Safe driving behavior	7074	49.7
Unsafe driving behavior	7165	50.3
Sickle cell anemia	n	%
No	13,789	96.8
Yes	450	3.2

**Table 2:** Predictors of sickle cell anemia among Saudis at primary healthcare settings in Riyadh (n = 14,239).

Predictors	OR	95% CI		AOR	95% CI	
		LL	UL		LL	UL
Age						
<50 years	1.00			1.00		
50 to 75 years	0.75	0.61	0.91	0.77	0.62	0.95
At least 75 years	0.53	0.39	0.72	0.62	0.44	0.86
Education						
Primary	1.00			1.00		
Up to High School	0.44	0.28	0.66	0.39	0.25	0.59
College/University	0.67	0.45	0.97	0.56	0.37	0.82
Gender						
Female	1.00			1.00		
Male	0.52	0.42	0.64	0.53	0.42	0.65
Marital status						
Single	1.00			NA		
Married	0.96	0.79	1.17			
Employment status						
Employed	1.00			NA		
Unemployed	1.14	0.94	1.37			
Insurance coverage						
No	1.00			1.00		
Yes	1.97	1.63	2.40	1.87	1.52	2.31

OR: odds ratio; AOR: adjusted odds ratio; 95% CI: 95% confidence interval; univariate analysis: *p* < 0.25 for inclusion; multivariable analysis: *p* < 0.05 for significance.

**Table 3:** Behavioral risk factors and comorbidities associated with sickle cell anemia (n = 14,239).

Predictors	OR	95%CI		AOR	95%CI		AOR	95%CI	
		LL	UL		LL	UL		LL	UL
Smoking									
No	1.00								
Yes	5.81	4.75	7.10	6.32	5.16	7.75	3.60	2.74	4.71
Physical activity									
No	1.00			1.00			1		
Yes	2.58	2.05	3.26	2.69	2.14	3.40	1.63	1.17	2.28
Fast food consumption									
No	1.00			1.00			1.00		
Yes	1.46	1.15	1.84	1.45	1.15	1.83	0.68	0.49	0.95
Obesity									
No	1.00			1.00			1		
Yes	26.22	21.36	32.18	27.04	21.94	33.31	1.86	1.39	2.49
Diabetes									
No	1.00			1.00			1		
Yes	4.59	3.77	5.60	6.32	5.12	7.81	1.39	1.01	1.91
Hypertension									
No	1.00			1.00			1		
Yes	9.00	7.42	10.91	12.65	10.28	15.56	1.70	1.23	2.35
Hypercholesterolemia									
No	1.00			1.00			1		
Yes	14.94	12.28	18.18	19.98	15.41	23.38	2.38	1.74	3.24
Heart disease									
No	1.00			1.00			1		
Yes	45.07	36.39	55.82	46.17	37.10	57.47	8.05	6.05	10.71

OR: odds ratio; AOR: adjusted odds ratio; 95% CI: 95% confidence interval. In the univariate analysis, the variables with a *p*-value of <0.25 were included for further consideration. In the multivariable analysis, a *p*-value of <0.05 was considered statistically significant. The adjusted odds ratios (AORs) were calculated in the multivariable analysis after controlling for age, sex, and mutual adjustment with the other behavioral risk factors and comorbidities.

## Data Availability

The original contributions presented in this study are included in the article. Further inquiries can be directed to the corresponding author.

## Abbreviations

The following abbreviations are used in this manuscript:

MDPI	Multidisciplinary Digital Publishing Institute
DOAJ	Directory of Open Access Journals
OR	Odds ratio
AOR	Adjusted odds ratio
CI	Confidence interval
